# Exploring Nutrient Deficiencies in Lettuce Crops: Utilizing Advanced Multidimensional Image Analysis for Precision Diagnosis

**DOI:** 10.3390/s25071957

**Published:** 2025-03-21

**Authors:** Jilong Xie, Shanshan Lv, Xihai Zhang, Weixian Song, Xinyi Liu, Yinghui Lu

**Affiliations:** College of Electrical and Information, Northeast Agricultural University, Harbin 150030, China; xiejl1979@163.com (J.X.); shanvshanwlv@163.com (S.L.); s221402010@neau.edu.cn (X.L.); 18846085348@163.com (Y.L.)

**Keywords:** multidimensional segmentation, image enhancement, image denoising, lettuce deficiency identification, real-time nutrient detection

## Abstract

In agricultural production, lettuce growth, yield, and quality are impacted by nutrient deficiencies caused by both environmental and human factors. Traditional nutrient detection methods face challenges such as long processing times, potential sample damage, and low automation, limiting their effectiveness in diagnosing and managing crop nutrition. To address these issues, this study developed a lettuce nutrient deficiency detection system using multi-dimensional image analysis and Field-Programmable Gate Arrays (FPGA). The system first applied a dynamic window histogram median filtering algorithm to denoise captured lettuce images. An adaptive algorithm integrating global and local contrast enhancement was then used to improve image detail and contrast. Additionally, a multi-dimensional image analysis algorithm combining threshold segmentation, improved Canny edge detection, and gradient-guided adaptive threshold segmentation enabled precise segmentation of healthy and nutrient-deficient tissues. The system quantitatively assessed nutrient deficiency by analyzing the proportion of nutrient-deficient tissue in the images. Experimental results showed that the system achieved an average precision of 0.944, a recall rate of 0.943, and an F1 score of 0.943 across different lettuce growth stages, demonstrating significant improvements in automation, accuracy, and detection efficiency while minimizing sample interference. This provides a reliable method for the rapid diagnosis of nutrient deficiencies in lettuce.

## 1. Introduction

Lettuce, a leafy vegetable crop cultivated globally, is often found to experience nutrient deficiencies during its growth cycle due to various environmental and management factors, commonly known as deficiency phenomena. Such deficiencies can lead to stunted growth, discoloration, and leaf deformation, subsequently reducing yield and nutritional value [1]. Traditionally, deficiency is diagnosed by experimentally analyzing soil and plant tissues, involving methods such as morphological diagnosis, chemical diagnosis, fertilization diagnosis, and enzymatic diagnosis methods [2], which, however, have not yet been extensively adopted in agricultural practices for their inability to balance accuracy, cost, detection speed, and universality [1]. Recent years have seen the gradual application of computer vision technology to diagnosing the nutritional status of crops. This technology is primarily categorized into methods based on chlorophyll content, multispectral and hyperspectral imaging technologies, and digital image analysis of abnormal features in plant parts [3]. These methods possess their own limitations despite their respective advantages. For instance, methods measuring chlorophyll content are challenged in the early stages of plant growth, where chlorophyll changes are not significant enough for timely deficiency detection. Multispectral and hyperspectral imaging techniques struggle with achieving high energy efficiency, resolution, signal-to-noise ratio, and stability. On the other hand, methods like digital image analysis of abnormal plant part features are known for their ability to rapidly and accurately identify deficiency signs based on changes in color and morphological abnormalities in leaf images. For example, Kang Xiaoyan predicted the nitrogen content of lettuce through principal component and multiple regression analyses [4], and Wei et al. rapidly graded the quality of external spinach through feature extraction [5]. Despite advances in image processing technology for crop nutritional diagnosis, the effectiveness of existing models and algorithms varies significantly across different crops, lacking universality. Existing studies mostly focus on mature leafy vegetables and tend to rely on single-leaf image analysis, typically involving destructive sampling and leading to resource wastage, and there is relatively scarce research on the identification of deficiency symptoms in lettuce crops [6].

Moreover, there was numerous noise interference in the process of transmitting lettuce images, which not only reduced the image quality but also affected the recognition results obtained based on these images. To reduce the impact of noise on image quality, a variety of denoising algorithms were developed [7]. Traditional adaptive median filtering algorithms and some improved median filtering methods achieved good denoising effects on software platforms, but these algorithms mostly employed serial sorting methods [8], which resulted in a massive computational amount and highly complex algorithms in the imaging process and thus extended image processing time [9].

However, appropriate image enhancement techniques were required to further enhance image quality after denoising, especially in cases where characteristic colors are lacking. Adaptive Histogram Equalization (AHE), developed by Stark and his colleagues, was aimed at effectively enhancing local contrast [10]. Yet, it was criticized for neglecting the overall image attributes, thereby affecting the quality and rendering effects of the entire image. The Average Weighted Histogram Equalization (AvHeq) method, proposed by Lin and his team [11], effectively preserved the brightness information of images but failed to take into account the color information. The Brightness Preserving Bi-Histogram Equalization (BBHE), developed by Kim et al., selected average brightness as the separation threshold while maintaining the overall brightness before and after enhancement [12]. This method may partially enhance images with a large dynamic range or uneven brightness. The Contrast Limited Adaptive Histogram Equalization (CLAHE) method, put forward by Lee J et al., was designed to limit the amplification of noise and excessive contrast enhancement in local areas caused by histogram equalization [13]. Subsequently, an improved CLAHE algorithm, featuring adaptive parameters T1 and T2, was introduced by Fang Danyang et al. [14]. This algorithm was effective in preventing the creation of artifacts yet exhibited deficiencies in handling global contrast issues.

Ultimately, image segmentation, as a key step in the processing flow, was utilized to divide the image into distinct regions with marked differences based on characteristics such as grayscale, color, texture, and shape, which facilitated subsequent feature extraction. While there were a variety of segmentation methods, there was a lack of a universal image segmentation algorithm. Methods still needed to be selected based on specific image features and application environments. Standard techniques, including region growing, area segmentation, and merging, were employed when images and their adjacent pixels were similar or even the same [15]. Edge tracking and detection were typically employed for image segmentation based on edge textures or sudden changes in grayscale values. The traditional edge detection techniques include the Canny algorithm, Sobel algorithm, Prewitt algorithm, and Log algorithm [16]. Among them, the Canny algorithm was widely recognized for its balanced performance, whose effectiveness in complex scenarios with a significant amount of noise, however, was limited. On this basis, the Canny algorithm underwent extensive research and adjustments by many experts, aiming to improve its efficacy in noisy environments. Yuanfeng Liu et al. focused on the target area and then employed an improved Canny operator for noise reduction, thereby enhancing the target detection accuracy [17], but accompanied by blurring image edges, potentially the omission of weak edges in the image. Wenbo Fu and his team combined CNN and Canny into the C-Canny algorithm, which improved the threshold positioning accuracy [18].

Despite progress in computer vision-based approaches for crop nutritional diagnosis, there remains a gap in the universality and robustness of segmentation algorithms tailored to practical applications [19,20]. Existing studies [21,22] primarily concentrate on isolated experimental conditions and lack comprehensive evaluations of real-world agricultural scenarios where variations in lighting, background complexity, and noise interference significantly impact image quality and recognition accuracy. Notably, while traditional image segmentation techniques such as region growing and edge detection have been widely used [23], their adaptability to varying lettuce deficiency symptoms under field conditions remains underexplored. Furthermore, recent deep learning-based segmentation models have demonstrated improved accuracy in plant phenotyping and disease detection; however, their computational complexity and dependence on extensive labeled datasets limit their feasibility for real-time applications in resource-constrained environments. Studies [24,25] utilizing convolutional neural networks (CNNs), such as U-Net and Mask R-CNN, have achieved high precision in leaf segmentation, yet they often struggle with processing speed and the generalization required for diverse lettuce cultivars. Additionally, research on integrating advanced edge detection techniques with adaptive segmentation methods remains limited, particularly in addressing the challenges of detecting subtle deficiency symptoms in lettuce canopies. Given these limitations, this study aims to bridge the gap by developing a Field-Programmable Gate Array (FPGA)-based nutrient deficiency detection system that incorporates an enhanced Canny edge detection algorithm and a multi-dimensional image analysis framework. By integrating adaptive thresholding and multi-directional gradient estimation, the proposed approach seeks to improve segmentation accuracy while maintaining computational efficiency.

In terms of noise reduction, a median filtering algorithm was proposed in this study based on a dynamic window histogram to effectively balance noise suppression and image detail preservation, significantly reducing the computational burden, and achieving rapid updates by utilizing a sliding window histogram technique or an adaptive. This algorithm was implemented on a hardware platform to compensate for the limitations of traditional PC serial processing in real-time processing. Furthermore, to address the potential issues of texture and detail loss, as well as over-enhancement caused by image enhancement algorithms, an improved CLAHE algorithm was introduced in this study. This improvement was manifested in three aspects: Firstly, an adaptive adjustment mechanism based on the overall brightness of the image was introduced to optimize the precision and effect of contrast enhancement while suppressing noise generated in the process; secondly, an adaptive smoothing distribution mechanism based on local histogram statistics was introduced, which dynamically adjusted contrast enhancement strategies according to local image features to preserve image details; and lastly, considering the need for image reconstruction, a 5 × 5 pixel neighborhood bicubic interpolation technique was employed to improve the detail preservation capability during image enlargement or resolution enhancement and thus enhance the overall visual quality of the image. Additionally, an adaptive global–local contrast enhancement algorithm (CLGCE) was proposed, which combines the advantages of Global Histogram Equalization (GHE) and the improved CLAHE technique. This algorithm achieved a greater balance in processing global brightness and local details. Finally, in order to better segment the characteristic color regions of lettuce, the Canny edge detection algorithm was enhanced to improve its accuracy and adaptability in complex image processing. Firstly, the gradient calculation of the algorithm was expanded by employing Sobel operators in eight directions, namely horizontal and vertical, as well as 45°, 135°, 180°, 225°, 270°, and 315° directions. This multi-directional gradient calculation helped to more comprehensively capture image edge information, especially the details in non-standard directions. Secondly, an improved method was introduced, considering gradients in diagonal directions and combining linear interpolation techniques, allowing image edges to be more accurately processed and estimated. Furthermore, an automatic threshold calculation strategy was proposed to dynamically compute thresholds based on image characteristics, significantly enhancing the algorithm’s adaptability to different image conditions. Based on these improvements, a novel multi-dimensional image analysis algorithm was further developed, specifically designed to accurately segment the defective areas of lettuce in complex environments. The high-precision characteristics of the improved multi-directional gradient adaptive threshold Canny edge detection algorithm were integrated into this algorithm, along with the efficiency of traditional threshold segmentation and the local adaptability of gradient-guided adaptive threshold segmentation. This integration was aimed at significantly enhancing the detection precision and processing efficiency of the defective areas of lettuce, even in variable and complex environments.

A reference for the development of deficiency analysis techniques in intelligent agriculture is provided by this lettuce nutrient deficiency detection method, which is characterized by its real-time, non-destructive, and automated nature. This research contributes to the advancement of intelligent agriculture by providing a real-time, non-destructive, and scalable solution for lettuce nutrient deficiency diagnosis, addressing key challenges in image noise reduction, segmentation accuracy, and adaptability across diverse cultivation environments.

## 2. Experiments and Materials

**Figure 1:**
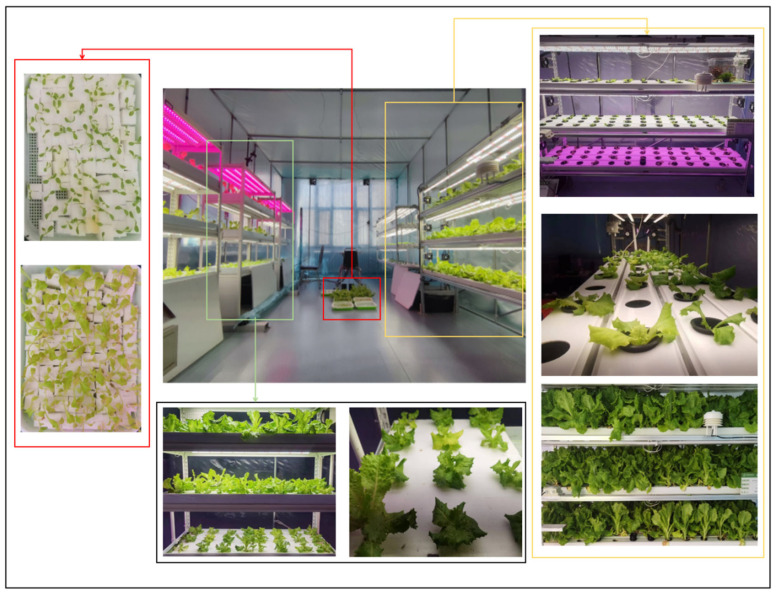
Experimental Scenario. The figure illustrates the hydroponic lettuce cultivation setup in a controlled plant factory environment. Different stages of plant development are depicted: the initial germination phase in seedling trays (top left), early seedling growth after 14 days (bottom left), and various stages of hydroponic lettuce growth in nutrient-rich solutions (right side and central image). The fully developed lettuce near harvest is shown in the bottom right section. The lighting system, hydroponic racks, and environmental control mechanisms used in the experiment are also visible.

The experiments on the nutrient deficiency of hydroponic lettuce were conducted from 10 March to 1 May 2024 in the Plant Factory of the College of Electrical and Information Engineering at Northeast Agricultural University. The experimental environment is shown in Figure 1.

**Figure 2:**
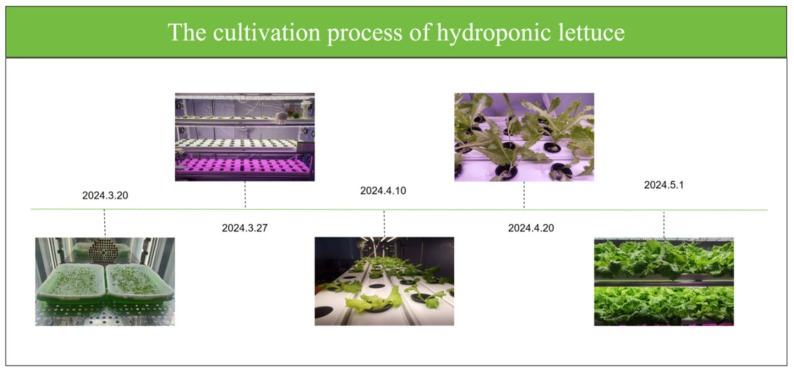
The cultivation process of hydroponic lettuce.

Lettuce seeds with good germination potential and healthy appearance were selected for the experiment and sown in seedling trays. During this stage, appropriate humidity and temperature conditions were maintained to promote seed germination. After meticulous cultivation for 14 days, the lettuce seedlings were transplanted into a hydroponic system. In the process of cultivating hydroponic lettuce, light intensity and nutrient solution concentration were identified as the primary environmental factors influencing growth, with a growth cycle of 40 days. A plant factory system was utilized during the experiment to uniformly regulate environmental parameters such as carbon dioxide concentration, temperature, airflow, and humidity, ensuring healthy growth under standardized conditions. In the hydroponic system, the pH of the nutrient solution was maintained between 5.5 and 6.5, and the electrical conductivity (EC value) was controlled within the range of 1400 to 1800 μS/cm. Red and blue light supplementation was applied in a timed manner, with the red-to-blue light ratio adjusted according to the growth stage of lettuce to ensure efficient photosynthesis and prevent abnormalities caused by insufficient light. Air circulation was achieved through a scheduled ventilation system that simulated natural conditions, providing an adequate carbon dioxide supply and preventing “tip burn”. The ambient temperature was controlled within the range of 18 to 22 °C, while the nutrient solution temperature was maintained at approximately 18 °C. Humidity level was kept between 40% and 85% to avoid excessive moisture, which potentially leads to rotting. Figure 2 illustrates the entire cultivation process of hydroponic lettuce, from sowing on 10 March 2024 to harvesting on 1 May 2024.

To study the effect of nutrient solution on the change in the color of the hydroponic lettuce canopy, cultivation groups with varying degrees of nitrogen, phosphorus, and potassium deficiency, along with the control group exhibiting normal growth, were designed. The nutrient solution used in this study was prepared according to the Hoagland formula, a widely used method for providing essential nutrients to plants in hydroponic systems [26]. This formula provides a balanced concentration of nutrients, including nitrogen, phosphorus, and potassium, which are critical for plant growth. The optimal concentrations of these nutrients for hydroponic lettuce cultivation are generally considered to be 15.000 mmol/L of nitrogen, 1.000 mmol/L of phosphorus, and 6.000 mmol/L of potassium, which were adopted in the control group of our experiment (Table 1). For the experimental groups, varying levels of these nutrients were systematically reduced to simulate deficiencies, with concentrations adjusted based on the severity of the deficiency: mild, moderate, or severe. The nutrient solution composition for each deficiency group was deliberately altered to reflect the targeted deficiency while maintaining a constant concentration of other essential nutrients to ensure plant growth under stress conditions. This approach allows a comparison of nutrient deficiency impacts across different plant growth stages and nutrient levels.

To ensure the accuracy and applicability of the experimental results, the experiment was conducted by subdividing the deficiency levels into mild, moderate, and severe so that subtle growth changes in plants could be captured under varying degrees of nutritional stress, thereby enabling the more precise diagnosis of deficiencies. Additionally, to enhance the reliability and statistical significance of the experimental data, a large number of samples were prepared for each deficiency level. The hydroponic lettuce sample data collected in this experiment will provide key sample evidence for establishing a real-time diagnosis system for nutrient deficiencies in hydroponic lettuce. The detailed data are listed in Table 2.

## 3. Optimization of Lettuce Canopy Image Processing Algorithm

As a comprehensive image analysis framework, the Lettuce Canopy Nutrient Deficiency Diagnostic Integrated Algorithm was aimed at providing an exact and thorough assessment of the nutritional status of hydroponically grown lettuce. With the integration of multiple advanced image processing sub-algorithms, complex growth environments were effectively coped with, thereby allowing for the accurate identification and analysis of the nutrient levels in lettuce. This method enhances the assessment precision and reliability and optimizes data processing.

### 3.1. Dynamic Window Histogram Median Filtering Algorithm

Noise will be inevitably generated during the acquisition, transmission, and processing of lettuce video images due to various factors such as electrical fluctuation, which will not only degrade image quality but also interfere with the detection of nutrient deficiencies in lettuce. Traditional median filtering performs well in suppressing random noise, especially “salt-and-pepper” noise, but its limitations are also evident. These include high computational complexity, significant hardware resource consumption, and reduced contrast, particularly when processing high-resolution images.

To address these issues, this study proposes an improved dynamic window histogram median filtering algorithm based on traditional median filtering. The algorithm employs an adaptive window adjustment mechanism based on local variance to dynamically select window size and achieve a balance between noise suppression and detail preservation. The window size was automatically reduced to protect critical details in high-variance areas (such as image edges and detail-rich regions), while appropriately enlarged to effectively remove noise in low-variance areas (such as smooth regions). Furthermore, the sliding window histogram technique ensures that window updates require only adjustments to the frequencies of incoming and outgoing pixels, eliminating the need to re-sort all pixels within the window, which significantly reduces computational overhead.

Compared to traditional dynamic filters, such as adaptive median filtering and Gaussian filtering, the proposed method demonstrates superior adaptability and flexibility. While adaptive median filtering helps to gradually expand the window size and remove salt-and-pepper noise, it lacks specificity when addressing complex image details. Gaussian filtering, which relies on fixed weight distributions for smoothing, often leads to blurring edges and is less effective in preserving details. The method proposed in this study not only flexibly adapts to the characteristics of different image regions but also significantly enhances the real-time processing efficiency. In practice, a 5 × 5 local window was defined for each pixel in the image, and the variance within each window was calculated, as illustrated in Equation (1),

(1) $$Var\left(x,y\right)=\frac{1}{N}\sum_{i,j\in W}{\left(I\left(i,j\right)-\mu \right)}^{2},$$

where $I\left(i,j\right)$ is the value of the pixels in the window, μ is the average value of the pixels in the window, and N is the number of pixels in the window.

The needs of different regions were adapted to by analyzing variance values. In areas where the local variance was higher than the threshold, the size of the filtering window was reduced to protect image details and edges. In areas where the local variance was below the threshold, the filtering window size was maintained to effectively remove noise. For each pixel point, the window size was chosen based on its local variance, as illustrated in Equation (2),

(2) $$W_{selected}\left(x,y\right)=\left\{\begin{array}{c} 3\times 3\;if\;Var\left(x,y\right)>\theta \\ 5\times 5\;if\;Var\left(x,y\right)\leq \theta \end{array}\right.,$$

where *θ* represents the set variance threshold.

Furthermore, for each color channel, an individual histogram H was constructed at the initial position of the adaptive window. The frequency of pixel values in the window for the respective color channel was recorded in each histogram. Then, for each color channel, a cumulative histogram was calculated, as illustrated in Equation (3),

(3) $$C\left(v\right)=\sum_{i=0}^{v}H\left(i\right),$$

where *H*(*i*) represents the frequency of pixel values equal to *I* in the corresponding channel.

As the window slid over the image pixel by pixel, only the counts of pixels entering and leaving the window in the histogram were updated. The count of the pixels leaving the window decreased, while that of the pixels entering the window increased. The cumulative histogram was recalculated at each new window position based on the updated histogram, allowing the median of the window to be rapidly determined. In an n × n sized window, the position of the median, M, is shown in Equation (4).

(4) $$M=\frac{n^{2}}{2}.$$

For each color channel, the specific pixel value of the median was the one that made the cumulative histogram reach or exceed half the size of the window for the first time. The particular calculation is shown in Equation (5),

(5) $$M=min\left\{v|C\left(v\right)\geq M\right\},$$

where *M* is the value v that makes C reach or exceed half the size of the window for the first time.

**Figure 3:**
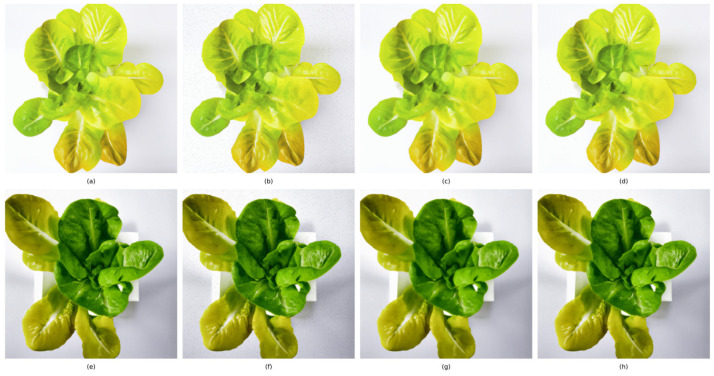
Comparison of noise reduction processing in lettuce images: (**a**,**e**) original image, (**b**,**f**) noisy image, (**c**,**g**) median filtered image, (**d**,**h**) adaptive median filtered image.

Ultimately, the computed median M was applied to each color channel, forming the new filtered image shown in Figure 3. Traditional median filtering is incapable of sharpening edges despite specific efficacy in noise removal, potentially leading to partial loss of image details. This phenomenon, manifested as relative blurriness compared to the original image, was evident, as shown in Figure 3c,g. In contrast, an improved adaptive median filtering algorithm was employed to finely process different parts of the image by adaptively adjusting the filtering window size, as shown in Figure 3d,h. In regions where there was a large amount of noise, a larger window was used for better effect, while in detail-rich regions, such as leaf veins and edges, a smaller window was used to maintain clarity.

### 3.2. Adaptive Combination of Global and Local Contrast Enhancement

Traditional Global Histogram Equalization (GHE) techniques are known for their capability to enhance the overall contrast of an image but often overlook local details. In comparison, Contrast Limited Adaptive Histogram Equalization (CLAHE) is focused more on local features of the image but can sometimes lead to excessive local enhancement. In this experiment, an adaptive combination of GHE and improved CLAHE techniques was employed to balance the effects of global and regional contrast in the image.

#### 3.2.1. Histogram Equalization

The goal of histogram equalization was to redistribute the pixel values of an image so that they uniformly covered the entire grayscale range as much as possible, thereby enhancing contrast and improving visual effects. During histogram equalization, only the V component in the HSV color space was processed. First, a histogram of the grayscale image was computed to obtain the pixel distribution of the original image. Then, the cumulative distribution function (CDF) for grayscale levels was calculated, as illustrated in Equation (6),

(6) $$CDF\left(r_{k}\right)=\sum_{j=0}^{k}P\left(r_{j}\right),$$

where $r_{k}$ is the grayscale level, and $P\left(r_{j}\right)$ is the probability of the grayscale level $r_{j}$.

Finally, each grayscale level of the original image was mapped to a new grayscale level, thereby achieving histogram equalization of the image. The mathematical expression for the mapping function is shown in Equation (7),

(7) $$G\left(r_{k}\right)=\left\lfloor \left(L-1\right)\cdot CDF\left(r_{k}\right)\right\rfloor ,$$

where L is the total number of grayscale levels, and $G\left(r_{k}\right)$ is the grayscale level after histogram equalization.

#### 3.2.2. Contrast Limited Adaptive Histogram Equalization

When the CLAHE (Contrast Limited Adaptive Histogram Equalization) algorithm was applied, the image was first divided into numerous small blocks, on each of which histogram equalization was performed independently.

(1) Average Brightness Calculation

(8) $$L_{avg}=\frac{1}{M\times N}\sum_{i=1}^{M}\sum_{j=1}^{N}I\left(i,j\right),$$

where M×N is the size of the image, and $\;I\left(i,j\right)$ is the brightness of the pixel at coordinate $\left(i,j\right)$.

(2) Dynamic Adjustment of Parameters

(9) $${clip\_Limit}_{adjust}=Base+Scale\times max\left(0,Threshold-L_{avg}\right),$$

where Base ensures contrast enhancement even in the case of high brightness, Scale is the scaling factor, and Threshold serves as the brightness threshold for determining the adjustment of parameters.

(3) Using ${clipLimit}_{adjust}$ for Contrast Limitation

(10) $$h_{R_{clipped}}\left(i\right)=min\left(h_{R}\left(i\right),{clipLimit}_{adjust}\right),$$

where $h_{R}\left(i\right)$ represents the number of pixels at grayscale level I in the original histogram, and $h_{R_{clipped}}\left(i\right)$ represents the number of pixels at grayscale level I in the histogram after contrast limitation.

(4) Adaptive Smoothing Allocation

Histograms for small image blocks were calculated, and excess pixels were identified. Weights were then assigned to each bin by identifying peaks and valleys, where valleys received higher weights to enhance contrast and peaks were assigned lower weights. The weight distribution can be calculated by Equation (11),

(11) $$w\left(i\right)=\frac{1}{1+exp\left(-\alpha \times \left(h\left(i\right)-Threshold\right)\right)},$$

where *w*(*i*) is the weight of the *i*th bin in the histogram and *h*(*i*) is the number of pixels in the *i*th bin of the histogram. *α* is an adjustment factor used to control the sensitivity in the weight distribution process. Based on these weights, excess pixels were proportionally distributed to each bin, as illustrated in Equation (12),

(12) $$h_{final}\left(i\right)=h_{clipped}\left(i\right)+excess\times \frac{w\left(i\right)}{\sum_{j=1}^{N_{bins}}w\left(j\right)},$$

where $h_{final}\left(i\right)$ is the histogram after distribution, and $h_{clipped}\left(i\right)$ is the histogram after the application of contrast limitation. If excess pixels remained after distribution, they could be allocated by traversing the bins again until all extra pixels had been distributed.

(5) Mapping Pixel Values

The original pixel values within each small block were mapped to new grayscale levels, as illustrated in Equations (13)–(15),

(13) $$DF\left(i\right)=\sum_{j=0}^{i}h_{final}\left(j\right),$$

(14) $${CDF}_{norm}\left(i\right)=\frac{CDF\left(i\right)-{CDF}_{min}}{M\times N-{CDF}_{min}}\times \left(MaxGrayLevel-1\right),$$

(15) $$I_{new}\left(x,y\right)={CDF}_{norm}\left[I_{original}\left(x,y\right)\right],$$

where $CDF\left(i\right)$ represents the cumulative number of pixels up to the gray level i, ${CDF}_{min}$ is the minimum value of CDF, MaxGrayLevel−1 is the highest gray level, $I_{original}\left(x,y\right)$ is the pixel value of the original image at coordinates $\left(x,y\right)$, and $I_{new}\left(x,y\right)$ is the new pixel value after mapping.

(6) Reconstruct Processed blocks

Using the bicubic interpolation algorithm with a 5 × 5 pixel neighborhood, the small blocks processed with CLAHE were arranged and combined according to their positions in the original image, as illustrated in Equations (16)–(18).

(16) $$K\left(u,v\right)=\left(1-{\left|u\right|}^{4}\right)\left(1-{\left|v\right|}^{4}\right).$$

(17) $$W\left(i,j\right)=K\left(\frac{i}{2},\frac{j}{2}\right).$$

(18) $$I_{new}\left(x,y\right)=\sum_{i=-2}^{2}\sum_{j=-2}^{2}I_{original}\left(x+i,y+j\right)\times W\left(i,j\right).$$

#### 3.2.3. Adaptive Global and Local Contrast Enhancement Algorithm

The specific implementation steps are described as follows:

(1) Calculate the Standard Deviation

(19) $$\sigma =\sqrt{\frac{1}{N}\sum_{i=0}^{N}{\left(I\left(i\right)-L_{avg}\right)}^{2}},$$

where σ is a statistical measure used to quantify the spread of brightness values in an image, reflecting the variation in brightness values relative to the mean.

(2) Calculate the Adaptive Weight

(20) $$\alpha =\frac{1}{1+e^{-k\left(L_{avg}-L_{0}\right)-m\left(\sigma -\sigma_{0}\right)}},$$

where k and m are parameters controlling the steepness of the curve, while $L_{0}$ and $\sigma_{0}$ are the preset thresholds for brightness and contrast, respectively.

(3) Result Fusion

(21) $$Z\left(i,j\right)=\alpha \cdot Z_{Global}\left(i,j\right)+\left(1-\alpha \right)\cdot Z_{Local}\left(i,j\right),$$

where $Z_{Global}\left(i,j\right)$ represents the pixel value at position $\left(i,j\right)$ after Global Histogram Equalization, and $Z_{Local}\left(i,j\right)$ represents the pixel value at the same position processed by the improved CLAHE. The adaptive weight coefficient α adjusts the contribution ratio between global equalization and local enhancement.

**Figure 4:**
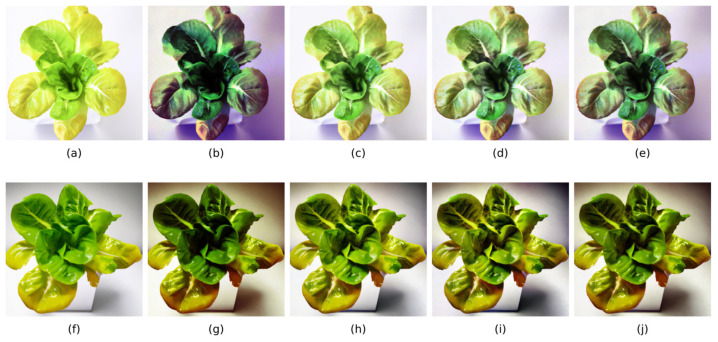
Lettuce image enhancement algorithm comparison chart: (**a**,**f**) original image, (**b**,**g**) histogram equalization, (**c**,**h**) CLAHE, (**d**,**i**) enhanced CLAHE, (**e**,**j**) combined adaptive.

The implementation effects of different algorithms are compared in Figure 4, where four other image enhancement methods improved the contrast compared to the original image. However, while histogram equalization increased the image contrast, its results were often overly saturated in terms of color representation, especially in the central area of the lettuce, making the colors unnatural and excessively intense, as illustrated in Figure 4b,g. In contrast, the Contrast Limited Adaptive Histogram Equalization (CLAHE) technique provided a more refined contrast enhancement, effectively avoiding color oversaturation in the lettuce, with richer details retained as the contrast was enhanced, as shown in Figure 4c,h. The enhanced CLAHE, by dynamically adjusting parameters, dynamically controlled contrast enhancement and adopted an adaptive smoothing strategy to optimize histogram distribution, thus avoiding color oversaturation while enhancing image contrast. Additionally, the implementation of a 5 × 5 pixel neighborhood bicubic interpolation algorithm resulted in smoother and more natural transitions of image details, which further helped to improve color balance and naturalness, as shown in Figure 4d,i. Lastly, the adaptive method that combined histogram equalization with enhanced CLAHE not only improved color fidelity but also maintained a natural feel of color distribution while enhancing contrast, as shown in Figure 4e,j.

### 3.3. Multidimensional Image Analysis Algorithm

The multidimensional image analysis algorithm combines the simplicity and efficiency of traditional threshold segmentation, the high precision of Canny edge detection, and the local responsiveness of the gradient-guided adaptive threshold segmentation algorithm. The advantage of this integrated approach is that the image region is initially delimited by threshold segmentation, and then the edge detection capability of the Canny algorithm is used to delimit the boundary accurately, and lastly, gradient-guided adaptive threshold segmentation is introduced to refine and adjust local thresholds in the segmentation process. This strategy has proved to be able to improve the precision in identifying boundaries between healthy and deficient tissues in lettuce images and significantly enhance the algorithm’s adaptability to complex changes in texture and illumination.

#### 3.3.1. Threshold Segmentation

In this study, all sample images were converted to the HSV color space prior to threshold segmentation. The three components of the HSV color space, namely hue, saturation, and value, were independent of each other. Therefore, the segmentation problem of a three-dimensional color space was transformed into three separate one-dimensional segmentation problems, as illustrated in Equation (22).

(22) $$T\left(x,y\right)=\left\{\begin{array}{c} 0\;if\;f\left(x,y\right)>T\\ 1\;if\;f\left(x,y\right)\leq T \end{array}\right..$$

#### 3.3.2. Multi-Directional Gradient Adaptive Threshold Canny Edge Detection Algorithm

**Figure 5:**
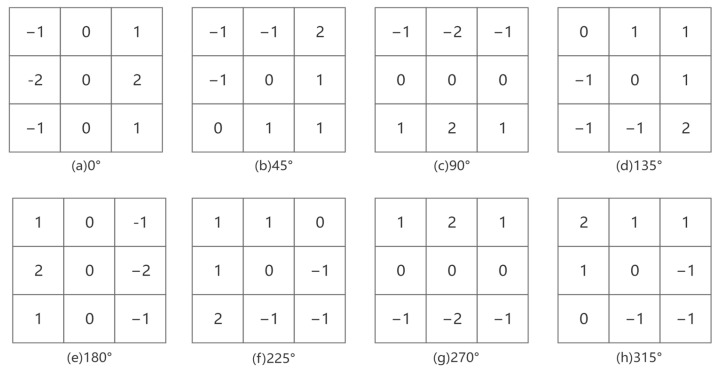
Sobel operator model for eight directions.

In traditional Canny algorithms, the Sobel operator is typically used to calculate gradients only in the horizontal and vertical directions, which limits their effectiveness in capturing edges that are diagonal or not aligned with these orientations. Moreover, if only these two directions are considered, edge orientations may be inaccurately detected, especially in cases where edge direction changes subtly. The Sobel operator is quite sensitive to noise in these two directions, thus potentially incorrectly identifying noise as edges. In addition to the traditional horizontal and vertical directions, gradient calculations in six other directions, namely 45°, 135°, 180°, 225°, 270°, and 315°, were introduced in this study. The Sobel operator was modified using various local 3 × 3 template operators for multi-directional gradient computation. These operators were convolved with the test image to derive gradient values in different directions. The enhanced Sobel operator model is detailed in Figure 5.

The gradient for each direction can be calculated by Equation (23),

(23) $$g_{\theta }\left(x,y\right)=\sum_{i=-1}^{1}\sum_{j=-1}^{1}G_{\theta }\left(i,j\right)*I\left(x+i,y+j\right),$$

where $g_{\theta }\left(x,y\right)$ represents the gradient at coordinates $\left(x,y\right)$ along a specific angle θ, and $G_{\theta }\left(i,j\right)$ is the corresponding Sobel kernel for the angle θ.

By combining the gradient components from each angle as per Equation (23), the total gradient magnitude and direction for each pixel were calculated, as shown in Equations (24) and (25), respectively,

(24) $$G=\sqrt{{G_{0}}^{2}+{G_{45}}^{2}+{G_{90}}^{2}+{G_{135}}^{2}+{G_{180}}^{2}+{G_{225}}^{2}+{G_{270}}^{2}+{G_{315}}^{2}},$$

(25) $$\theta \left(x,y\right)=\tan^{-1}\left(\frac{\sum_{\theta }g_{\theta }\left(x,y\right)\cdot \sin\theta }{\sum_{\theta }g_{\theta }\left(x,y\right)\cdot \cos\theta }\right),$$

where $\theta \in \left\{0^{{}^{\circ}},45^{{}^{\circ}},90^{{}^{\circ}},135^{{}^{\circ}},180^{{}^{\circ}},225^{{}^{\circ}},270^{{}^{\circ}},315^{{}^{\circ}}\right\}$. sin⁡θ and cos⁡θ were used to calculate the projections of the gradient components in the vertical and horizontal directions, respectively.

**Figure 6:**
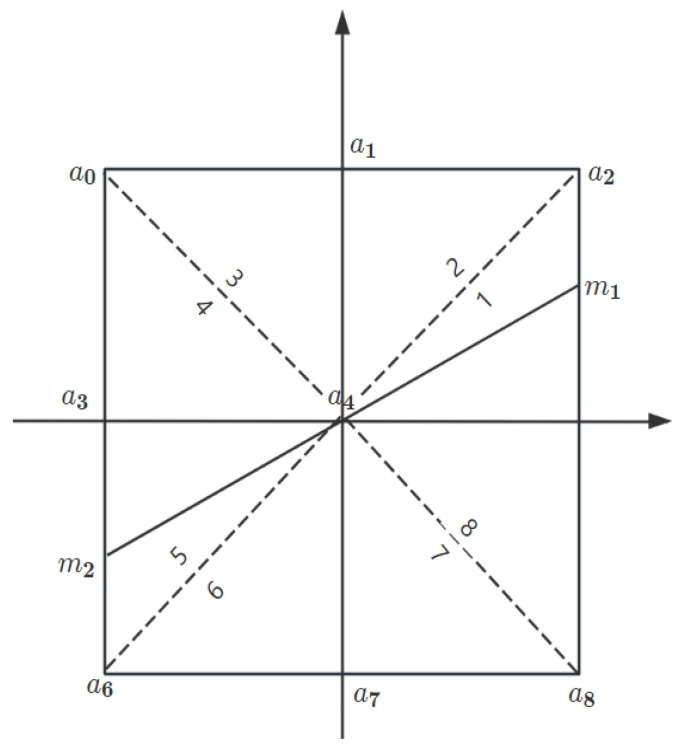
Gradient direction and the current pixel window.

By calculating Equation (24), the gradient magnitudes in various directions were precisely determined. Higher gradient magnitudes often indicated areas with significant brightness changes, marking potential edge locations. However, larger gradient magnitudes did not always accurately point to actual edges; they could sometimes originate from noise or other non-edge features in the image. To address this issue, the Canny edge detection algorithm employed a technique known as non-maximum suppression (NMS). Yet, traditional NMS methods had certain limitations when dealing with pixel values in the gradient directions, especially in handling gradients on diagonal lines. To overcome this drawback, an improved method that accounted for gradients in diagonal directions and applied linear interpolation techniques was introduced in our experiment, which enabled a more accurate estimation of gradient magnitudes at non-integer pixel positions. Specifically, the Sobel operation results underwent a 3 × 3 windowing process. Letting $a_{0}\sim a_{8}$ represent the 3 × 3 neighborhood pixel points in the current window, with $a_{4}$ being the central pixel of the current window and the vector from $m_{1}$ to $m_{2}$ denoting the gradient direction at the current pixel point $a_{4}$, we obtained Figure 6.

In Figure 6, the diagonal direction was the gradient direction of point $a_{4}$, indicating that the local maxima were distributed along this line. However, $m_{1}$ and $m_{2}$ were not precisely located in the integer domain. Therefore, linear interpolation was used to estimate the values of $m_{1}$ and $m_{2}$, further refining the edge localization. The mathematical expressions for calculating $m_{1}$ and $m_{2}$ are provided in Equations (26) and (27).

(26) $$f\left(m_{1}\right)=\frac{y}{x}*a_{2}+\left(1-\frac{y}{x}\right)*a_{5}.$$

(27) $$f\left(m_{2}\right)=\frac{y}{x}*a_{6}+\left(1-\frac{y}{x}\right)*a_{3}.$$

If the edge strength of the central pixel was not a local maximum, then the edge strength of that pixel was set to zero, as shown in Equation (28).

(28) $$Result=\left\{\begin{array}{c} 1,\left[a_{4}\geq f\left(m_{1}\right)\right]\;\mathrm{and}\;\left[a_{4}\geq f\left(m_{2}\right)\right]\\ 0,\mathrm{otherwise} \end{array}\right..$$

While non-maximum suppression in the Canny edge detection algorithm reduces false edges, there are still some problems: false edges are not eliminated, and image edges are not closed. The traditional Canny algorithm implemented a dual-threshold technique to reduce false edges and improve edge detection accuracy more effectively. This technique distinguished between strong and weak edges, enhancing the precision of edge detection. However, its obvious drawback was its reliance on two manually set thresholds. This static threshold setting often failed to adapt to a diverse range of image conditions, which limited the algorithm’s applicability and generalization capabilities. To address this issue, an automatic threshold calculation strategy was introduced in this study. This strategy dynamically calculated thresholds based on image characteristics, thereby significantly enhancing the adaptability of the Canny algorithm to various image conditions.

A gradient magnitude histogram was constructed based on the gradient magnitudes for each pixel in all directions, as obtained from Equation (24). This histogram used 95% of the gradient magnitude as the high threshold. The formula for calculating the high threshold is shown in Equation (29),

(29) $$T_{high}=Percentile\left(H_{gradient},95\%\right),$$

where $H_{gradient}$ is the gradient magnitude histogram, and 95% is the selected percentile threshold. The low threshold was selected based on a certain proportion of the high threshold, and the formula for calculating the low threshold is shown in Equation (30),

(30) $$T_{low}=Ratio\times T_{high},$$

where $H_{gradient}$ is the high threshold previously determined from the gradient histogram, and Ratio is the coefficient that determines what multiple of the high threshold the low threshold will be.

**Figure 7:**
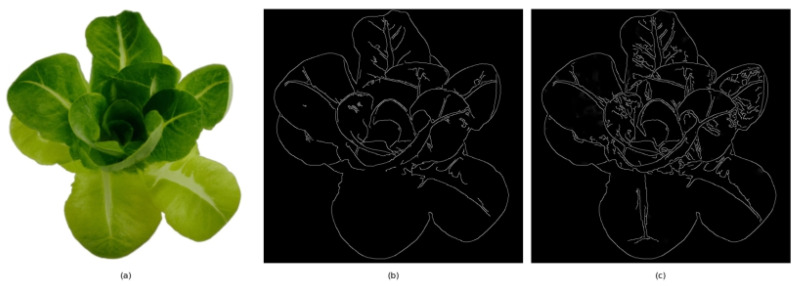
Comparison of edge detection results before and after improvement: (**a**) original image, (**b**) traditional Canny edge detection algorithm, (**c**) improved Canny edge detection algorithm.

The improved edge detection technique was applied to images of lettuce and compared with traditional methods. Figure 7 shows a clear difference in edge detection quality between the two processes. The original image on the left displays the lettuce in its natural form. In contrast, the middle image shows the results of using the standard Canny edge detection algorithm, where the leading edges were identified but lack ideal continuity and noise control. In contrast, the image on the right, employing the improved edge detection algorithm, demonstrated high-definition edges and greater coherence. Additionally, there was a significant improvement in noise suppression and edge localization accuracy, especially in depicting the details of lettuce leaf tips and edges. This experimental result validated the effectiveness of the improved algorithm, which significantly enhanced edge detection performance by integrating multi-directional gradient analysis, improved non-maximum suppression, and adaptive thresholding strategies, allowing the algorithm to identify and track actual edges in the image more accurately.

#### 3.3.3. Gradient-Guided Adaptive Threshold Segmentation Algorithm

The calculation of the adaptive threshold and gradient magnitude analysis together formed the core of the gradient-guided adaptive threshold segmentation method. This combination enhanced the algorithm’s flexibility and improved its ability to accurately segment different image regions. Initially, the Sobel operator was used to calculate the gradient of the image after Canny edge detection, with the gradient results obtainable according to Equation (24). Subsequently, the adaptive threshold calculation focused on adjusting the threshold according to local image characteristics. The original image was divided into multiple small blocks, with the threshold for each block determined by the pixel intensity characteristics within that area. Specifically, a weight $w\left(x,y\right)$ was assigned based on the distance from each pixel point $\left(x,y\right)$ to the center of the region $R_{i}$. The equation for calculating the weight is expressed as follows:

(31) $$w\left(x,y\right)=\frac{1}{1+d\left(\left(x,y\right),\left(x_{c},y_{c}\right)\right)},$$

where $d\left(\left(x,y\right),\left(x_{c},y_{c}\right)\right)$ represents the distance between the pixel point $\left(x,y\right)$ and the center of the region $\left(x_{c},y_{c}\right)$. For each region $R_{i}$, the adaptive threshold $T_{i}$ was calculated using weighted average, whose formula can be specifically expressed as follows:

(32) $$T_{i}=\frac{\sum_{\left(x,y\right)\in R_{i}}w\left(x,y\right)\cdot I\left(x,y\right)}{\sum_{\left(x,y\right)\in R_{i}}w\left(x,y\right)},$$

where $\sum_{\left(x,y\right)\in R_{i}}w\left(x,y\right)\cdot I\left(x,y\right)$ is the sum of the weighted intensities of all pixels in region $R_{i}$, and the denominator $\sum_{\left(x,y\right)\in R_{i}}w\left(x,y\right)$ is the total sum of weights in the region. For each pixel point $\left(x,y\right)$ in the image, the obtained gradient magnitude was compared with the adaptive threshold $T_{i}$. If a pixel point $\left(x,y\right)$ had a gradient magnitude exceeding the threshold of its region, then it was marked as foreground. This is represented by Equation (33).

(33) $$S\left(x,y\right)=\left\{\begin{array}{c} 1,if\;G_{\left(x,y\right)}>T_{i}\;\mathrm{for}\;\mathrm{all}\;\left(x,y\right)\in R_{i}\\ 0,\;\;\;\mathrm{otherwise} \end{array}\right.$$

**Figure 8:**
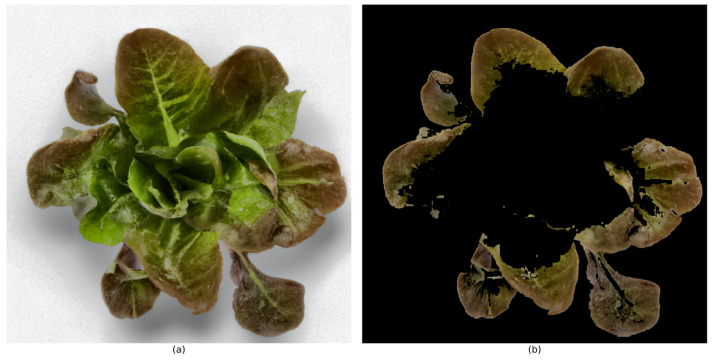
Multidimensional segmentation results of lettuce images: (**a**) unsegmented lettuce leaves, (**b**) segmented lettuce leaves.

In this study, a number of algorithms were efficiently integrated by a multidimensional image analysis method, offering an efficient, precise, and responsive image segmentation solution. Initially, traditional threshold segmentation was employed to rapidly delineate the primary areas of the image, which laid a foundation for subsequent processing. The introduction of Canny edge detection, with its high-precision edge localization capability, further clarified the boundaries of these areas, ensuring their clarity and continuity. Finally, the gradient-guided adaptive threshold segmentation algorithm optimized this process by adjusting thresholds based on local image characteristics, which enhanced the representation of image edges and texture details. Compared to traditional threshold segmentation methods, this integrated approach is better adapted to regional variations in the image. The multi-dimensional segmentation results demonstrated the fine segmentation of the nutrient-deficient areas in lettuce, as shown in Figure 8.

## 4. Lettuce Real-Time Diagnostic System

**Figure 9:**
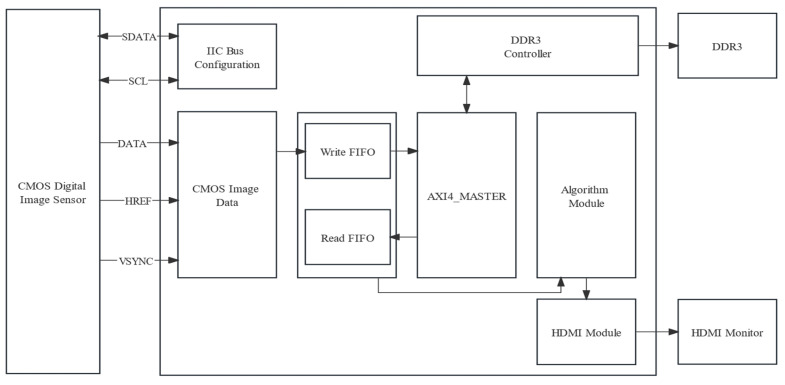
System overall block diagram.

The hardware architecture constructed in this study was divided into four modules, namely a real-time image capture module, a storage module, an algorithm processing module, and an HDMI display module. The overall framework of the system is depicted in Figure 9. Each module had its own specific role: the image capture module, the storage module, the algorithm processing module, and the HDMI display module were, respectively, dedicated to real-time image acquisition, image data storage, nutritional deficiency analysis on the captured images, and displaying processed images in real-time on an HDMI screen.

**Figure 10:**
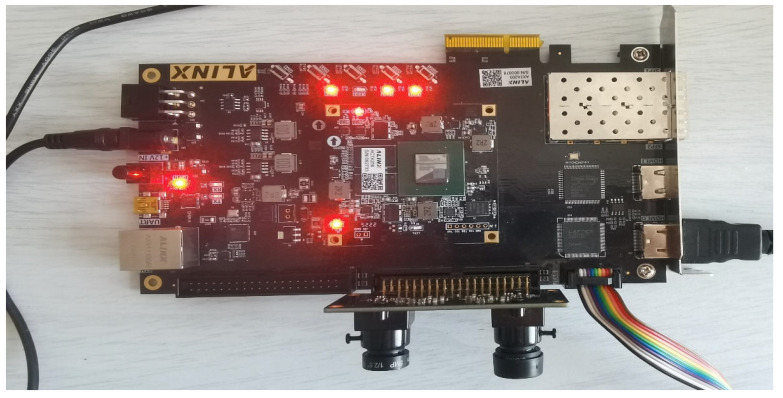
Main controller physical diagram.

The ARTIX-7 series 200T XC7A200T-2FBG484I FPGA device from XILINX (San Jose, CA, USA) was used by the master controller to coordinate the data conversion of the OV5640 image sensor and SDRAM memory, drive the HDMI display screen, and implement algorithmic processing of image data, as shown in Figure 10.

To enhance the reliability of classification results, the system integrated a multi-stage classification process. The classification procedure did not involve traditional machine learning training, validation, or testing stages but instead relied on predefined decision rules to optimize nutrient deficiency detection. Image data collected from the real-time capture module were processed to extract characteristic color features and construct a feature space. A decision-tree-based classification model was then applied to categorize lettuce nutrient deficiency levels based on these extracted features. The classification decisions were determined using predefined criteria derived from labeled lettuce images with verified nutrient deficiency conditions, ensuring consistent and interpretable outcomes.

The classification performance of the system was quantitatively evaluated using precision, recall, and F1-score metrics. These metrics were computed based on the confusion matrices, which captured the system’s ability to correctly identify nutrient deficiencies at different growth stages. The confusion matrices were constructed by comparing system predictions with ground truth labels obtained from expert assessments, and classification errors were analyzed based on the non-diagonal elements of the matrices. Statistical validation of classification performance was conducted using a chi-square test to assess the significance of misclassifications, ensuring that the conclusions drawn from the results were robust and reliable. The classification errors mentioned in line 658 were calculated by determining the proportion of misclassified instances in relation to the total number of classified samples. Misclassification trends were analyzed to identify potential sources of error, such as variations in lighting conditions and leaf texture inconsistencies. These insights contributed to the refinement of the algorithm and guided the future development of the system toward incorporating additional feature extraction techniques, including texture and shape analysis, to enhance classification accuracy.

**Figure 11:**
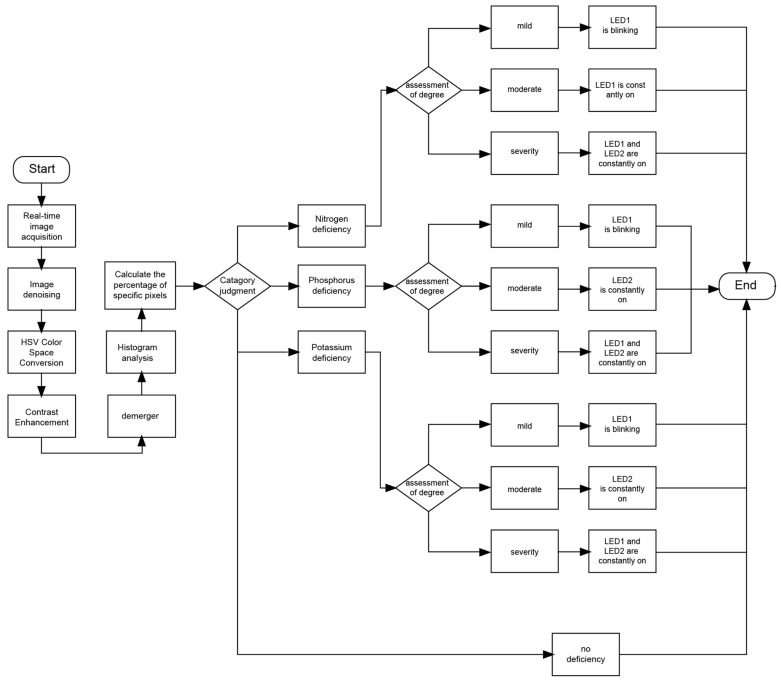
System flowchart.

The workflow is described as follows. After a power reset, the system was initiated, and each module entered the initialization phase. This phase was managed by the central control FPGA chip, which not only drove the camera module, storage module, algorithm module, and HDMI module but also provided the necessary clock signals and configured the internal registers of each module. After initialization, the CMOS sensor collaborated with the FPGA and captured RGB video data using line and field signals. And these data were transferred to the FPGA and buffered in DDR3 memory and then further processed in the algorithm module. The primary task was to denoise the captured images of the lettuce canopy, convert the image data format from RGB565 to HSV mode, and enhance contrast for better detail information. Then, the characteristic colors and background of the lettuce canopy were precisely segmented, and the segmented images were displayed in real-time on an HDMI monitor. To classify the nutrient deficiencies, a histogram-based classification model was implemented. This model analyzed the distribution of pixel values within each characteristic color region, applying a threshold-based decision rule to distinguish among different nutrient deficiency levels. The classification process involved constructing feature vectors based on the pixel frequency distribution and mapping them to predefined deficiency categories. The system subsequently conducted histogram analysis on the segmented pixel values, classified them based on the frequency of the predominant pixel values in each characteristic color, and determined the nutritional deficiencies of the lettuce by lighting up different LED lights. The workflow diagram of this study demonstrated each step from data collection to the display of final results, as shown in Figure 11.

## 5. Experimental Results and Analysis

**Figure 12:**
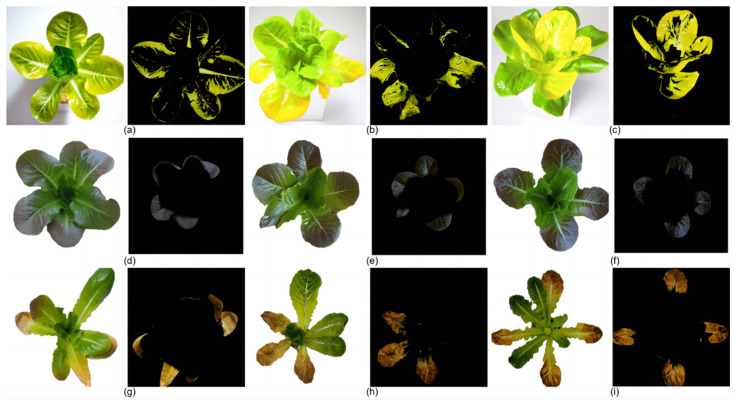
Segmentation effect of characteristic colors in lettuce leaves by the real-time diagnostic system. (**a**) Segmentation of colors in a mildly nitrogen-deficient leaf, (**b**) segmentation of colors in a moderately nitrogen-deficient leaf, (**c**) segmentation of colors in a severely nitrogen-deficient leaf, (**d**) segmentation of colors in a mildly phosphorus-deficient leaf, (**e**) segmentation of colors in a moderately phosphorus-deficient leaf, (**f**) segmentation of colors in a severely phosphorus-deficient leaf, (**g**) segmentation of colors in a mildly potassium-deficient leaf, (**h**) segmentation of colors in a moderately potassium-deficient leaf, (**i**) segmentation of colors in a severely potassium-deficient leaf.

In this study, in the real-time nutrient deficiency detection process in mature lettuce leaves, the characteristic colors of nutrient-deficient areas were accurately segmented from the background. The nutrient deficiency segmentation results for mature lettuce in this study are depicted as follows: Figure 12a–c for the N-deficient group, Figure 12d–f for the P-deficient group, and Figure 12g–i for the K-deficient group.

To ensure the generalization capability of the proposed system, the dataset was collected from multiple hydroponic lettuce cultivation setups under varying environmental conditions, including different lighting intensities and background variations. Additionally, data augmentation techniques, such as brightness normalization and contrast enhancement, were applied to mitigate biases associated with specific acquisition conditions. However, future studies will concentrate on validating the system on an independent dataset obtained from external sources to further assess its robustness in diverse practical scenarios. All reported accuracy, precision, recall, and F1-score values represent the mean of multiple trials conducted across different lettuce growth stages. Standard deviations were calculated and are provided in Table 3 to illustrate the variability in system performance. A K-fold cross-validation approach (K = 5) was employed to estimate the mean and standard deviations of all reported metrics, ensuring a more reliable performance evaluation. In cases where K-fold cross-validation was not applicable, alternative resampling methods were used to assess the stability of the diagnostic outcomes. To further validate the reproducibility of the results, each experiment was conducted multiple times, with the number of trials per growth stage and nutrient deficiency level recorded. Confidence intervals were calculated for key performance metrics to provide statistical insights into the system’s reliability. These results confirm that the system maintains high accuracy and consistency in diagnosing nutrient deficiencies in lettuce across different growth stages.

To ensure the reliability of the results, statistical measures including mean, standard deviation, and confidence intervals were calculated for the accuracy values across multiple trials. These statistical parameters provide a more robust assessment of the system’s performance by quantifying variations in detection accuracy under different conditions. Each experiment was conducted across multiple trials to mitigate the effects of variability in image acquisition and processing. A K-fold cross-validation approach was employed to assess the system’s generalizability and stability, ensuring that the reported performance metrics are not overly optimistic due to a specific set of test samples. To further validate the effectiveness of the proposed diagnostic system, a comparative analysis was conducted against existing lettuce nutrient deficiency detection methods. Performance metrics such as precision, recall, and F1-score were benchmarked against alternative image-based diagnostic techniques, and the observed differences were statistically analyzed using paired *t*-tests (or ANOVA, if applicable) to determine the significance of performance improvements.

**Figure 13:**
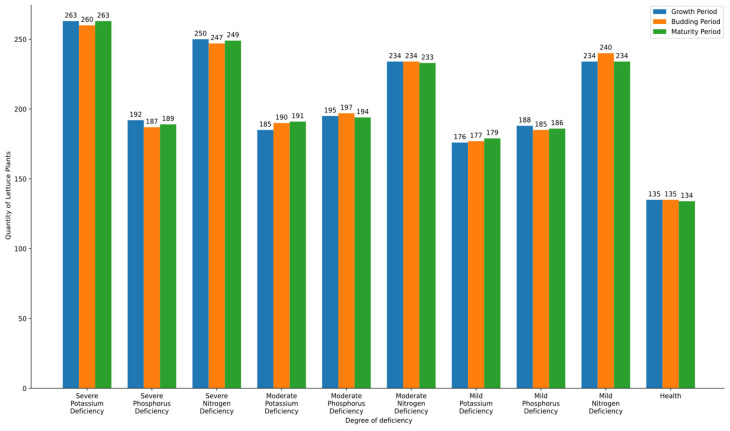
Nutrient deficiency identification chart for lettuce at different growth stages.

After multiple experiments, the number of lettuce plants identified by the system at different growth stages and varying levels of nutrient deficiencies was collected and organized. To evaluate the effectiveness of the proposed model, a comparison with existing classification techniques, such as machine learning-based classifiers (e.g., SVM, CNN), should be conducted. However, in this study, a hardware-optimized histogram analysis approach was prioritized to ensure real-time processing on FPGA. Future research will explore the integration of alternative classification techniques to assess performance differences and further improve diagnostic accuracy. To clearly illustrate the identification of nutrient deficiencies in lettuce at different time stages, a bar graph was created, as shown in Figure 13. Table 3 further presents the accuracy of the system in identifying lettuce plants with different levels of nutrient deficiencies across various growth stages.

**Figure 14:**
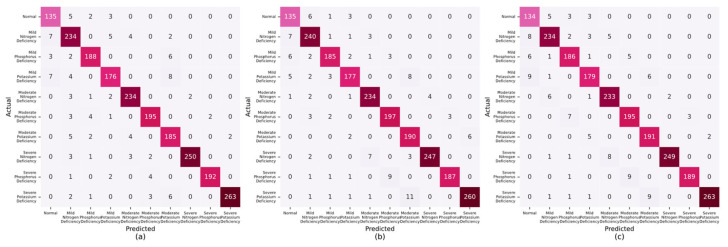
Confusion matrix analysis for lettuce identification at different growth stages: (**a**) the growing period, (**b**) the budding period, (**c**) the maturity period.

A comparative analytical approach was adopted to assess the efficacy of the proposed lettuce nutrient deficiency detection system. This process entailed aligning the nutrient deficiencies that were recognized by the system with the genuine deficiencies noted in lettuce at different growth stages. The confusion matrices, which were used to illustrate the accuracy of lettuce identification in the growing period, budding period, and maturity period, are presented in Figure 14a–c. Elevated values along the principal diagonal of these matrices underscored the system’s proficiency in accurately identifying instances. In contrast, the values in the non-diagonal elements highlighted discrepancies between the nutrient-deficient states predicted by the system and the actual nutrient-deficient states, clearly indicating the categories of misclassification. These findings corroborated the system’s robust capability to discern three distinct levels of nutrient deficiencies in lettuce across diverse growth stages.

The real-time diagnostic system developed in this study demonstrated significant improvements in efficiency, accuracy, and real-time processing compared to existing nutrient deficiency detection methods [24,25]. Traditional approaches, such as manual inspection or non-automated image processing techniques, often suffer from subjectivity, time inefficiency, and susceptibility to environmental factors, such as lighting variations. Some recent studies have explored machine learning-based classification methods for nutrient deficiency detection; however, these typically rely on offline processing, require extensive labeled datasets, and may not provide real-time diagnostic capabilities.

**Figure 15:**
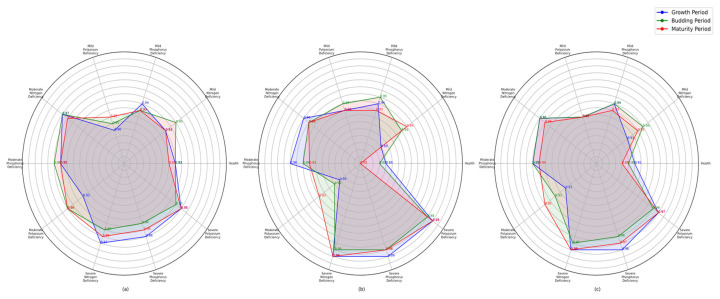
Nutrient deficiency diagnosis performance in lettuce at different growth stages: (**a**) radar chart of precision for lettuce nutrient deficiency detection, (**b**) radar chart of recall for lettuce nutrient deficiency detection, (**c**) radar chart of F1 score for lettuce nutrient deficiency detection.

In contrast to conventional methods, this study employed an FPGA-based hardware-accelerated architecture, enabling parallel processing and significantly reducing computational latency. The system integrated advanced image processing techniques, including dynamic window algorithms and contrast enhancement, ensuring robustness in diverse environmental conditions. Additionally, while deep learning-based approaches provide promising classification accuracy, they are often computationally intensive and require substantial training data. In contrast, the proposed system efficiently processes real-time image data without necessitating extensive model training, making it more suitable for practical agricultural applications. Although direct comparison with existing methods is challenging due to variations in experimental conditions, dataset characteristics, and evaluation metrics, the proposed system demonstrates high reliability in detecting nutrient deficiencies across different growth stages, achieving an average precision of 0.944, recall of 0.943, and F1 score of 0.943. As shown in Figure 15, the nutrient deficiency diagnosis performance in lettuce at various growth stages is illustrated: Figure 15a radar chart of precision for lettuce nutrient deficiency detection, Figure 15b radar chart of recall for lettuce nutrient deficiency detection, and Figure 15c radar chart of F1 score for lettuce nutrient deficiency detection. These performance metrics suggest that the system provides a viable alternative to both traditional visual assessment and data-intensive machine learning approaches, striking a balance between accuracy, real-time processing, and practical applicability in hydroponic farming. Furthermore, the effect of the plant’s growth stage on the accuracy and quality of nutrient deficiency detection was considered. It was observed that the system’s performance varied slightly across the different growth stages, with the highest accuracy typically achieved during the budding and maturity periods. The precision of deficiency detection in the growing period was slightly lower, possibly due to the morphological and physiological changes in the plant at this stage. This suggests that the growth stage could influence the system’s ability to detect nutrient deficiencies, especially in the early stages of lettuce development. Future research could explore the impact of specific growth stages in greater detail to optimize the detection process for various developmental phases. 

## 6. Conclusions

This study fully utilized the parallel processing capabilities of FPGA (Field-Programmable Gate Array), combined with multidimensional image analysis algorithms, optimized image enhancement algorithms, and image denoising techniques, to propose a novel real-time diagnostic system for nutrient deficiencies in hydroponically cultivated lettuce. The system was able to precisely identify nutrient deficiencies of lettuce across different growth stages, with an average precision of 0.944, a recall of 0.943, and an F1 score of 0.943, underscoring its high diagnostic efficiency and accuracy, which satisfied the requirements for automation, real-time processing, and non-destructive detection.

The main innovations of this study are reflected in the following aspects: First, the system employed real-time image processing methods to significantly enhance image processing efficiency and reduce computational latency through hardware-accelerated algorithms, thus providing an efficient solution for the real-time diagnostic needs of agricultural applications. Second, by leveraging the FPGA-based parallel processing architecture, the system enabled efficient analysis of multidimensional image features, which not only extracted color features but also focused on edge details and local variations in the image, making it capable of precisely detecting subtle nutrient-deficient regions under various environmental conditions. Furthermore, the proposed dynamic window algorithm effectively handled noise and lighting variation, ensuring the robustness of the system in complex real-world scenarios.

Nevertheless, this study has certain limitations. Specifically, the system still heavily relies on color features in some cases, which are inherently susceptible to the influence of lighting conditions and leaf aging, potentially leading to classification errors. Future research will focus on further expanding the range of features, such as incorporating information on leaf texture, shape, and light reflection properties, while integrating multi-angle machine learning techniques to develop a more comprehensive and accurate diagnostic model for nutrient deficiencies.

Although this study demonstrated the feasibility and effectiveness of the proposed system in identifying nutrient deficiencies in lettuce, there remains room for further exploration in future research. In particular, quantitative assessments of image quality, such as peak signal-to-noise ratio (PSNR) and structural similarity index (SSIM), will be considered to evaluate the impact of image enhancement, noise reduction, and edge detection techniques on overall image clarity and diagnostic accuracy. Moreover, further experimental comparisons with other existing systems will provide a more comprehensive understanding of the proposed system’s performance in various real-world conditions. Future research will also concentrate on extending the analysis to include texture-based features and multi-angle imaging techniques, which may enhance classification robustness and reduce dependency on color features that are sensitive to environmental factors, such as lighting and leaf aging.

By proposing an innovative, hardware-accelerated real-time diagnostic system, this study provides an efficient and practical solution for detecting nutrient deficiencies in hydroponically cultivated lettuce and offers valuable insights and directions for advancing precision agricultural technologies, paving the way for more intelligent and sustainable agricultural practices.

## Figures and Tables

**Table 1:** Comparison of nitrogen, phosphorus, and potassium nutrient concentration settings.

Group	Category	Nitrogen Content mmol/L	Phosphorus Content mmol/L	Potassium Content mmol/L
Experimental Group	Mild Nitrogen Deficiency	11.244	1.000	6.000
	Moderate Nitrogen Deficiency	7.496	1.000	6.000
	Severe Nitrogen Deficiency	3.748	1.000	6.000
	Mild Phosphorus Deficiency	15.000	0.750	6.000
	Moderate Phosphorus Deficiency	15.000	0.500	6.000
	Severe Phosphorus Deficiency	15.000	0.250	6.000
	Mild Potassium Deficiency	15.000	1.000	4.507
	Moderate Potassium Deficiency	15.000	1.000	3.005
	Severe Potassium Deficiency	15.000	1.000	1.5002
Control Group	Healthy	15.000	1.000	6.000

**Table 2:** Hydroponic lettuce sample data summary table.

Period	Healthy Plants	Mild Deficiency	Plants	Moderate Deficiency	Plants	Severe Deficiency	Plants
		N	252	N	242	N	259
Growth Stage	145	P	199	P	205	P	199
		K	195	K	198	K	275
		N	252	N	242	N	259
Bolting Stage	145	P	199	P	205	P	199
		K	195	K	198	K	275
		N	252	N	242	N	259
Maturity Stage	145	P	199	P	205	P	199
		K	195	K	198	K	275

**Table 3:** Number of lettuce plants at different growth stages and nutrient deficiencies.

Stage	Healthy Plants	Accuracy	Mild Deficiency	Plants	Accuracy	Moderate Deficiency	Plants	Accuracy	Severe Deficiency	Plants	Accuracy
			N	252	92.8%	N	242	96.6%	N	259	96.5%
Growth Period	145	93.1%	P	199	94.4%	P	205	95.1%	P	199	96.4%
			K	195	90.2%	K	198	93.4%	K	275	95.6%
			N	252	95.2%	N	242	96.6%	N	259	95.3%
Budding Period	145	93.1%	P	199	94.4%	P	205	96.0%	P	199	93.9%
			K	195	90.7%	K	198	95.9%	K	275	94.5%
			N	252	92.8%	N	242	96.2%	N	259	96.1%
Maturity Period	145	92.4%	P	199	93.4%	P	205	94.6%	P	199	94.9%
			K	195	91.7%	K	198	96.4%	K	275	95.6%

## Data Availability

The original data, including implementation code and sample data, presented in the study are openly available at https://github.com/lvss88 (accessed on 23 January 2025).

## References

[1] Xu S., Lin W., Wu W., Zhao H. (2015). Image Diagnosis of Rapeseed Deficiency Based on Color Features. J. Chin. Oil Crop Sci..

[2] Zhang J., Tian H., Li Z., Li F., Shi S. (2018). Nitrogen Nutrition Monitoring of Sugar Beet Canopies Based on Digital Camera Images. Trans. Chin. Soc. Agric. Eng..

[3] Zhang K., Zhang A., Li C. (2016). Diagnosis of Rapeseed Leaf Deficiency Based on Color Histogram in HSV Space. Trans. Chin. Soc. Agric. Eng..

[4] Gu P., Sun J., Kang X.-Y., Yi M., Li X.-Q., Xue P., Li R. (2013). A Formal Metal-Free N-Arylation via the Schmidt Reaction of Aromatic Aldehydes with an Azido Amine. Org. Lett..

[5] Wei W., Xing Y., Li Y., Peng Y., Zhang W. (2018). Online Detection and Grading System for External Quality of Leafy Vegetables Suitable for Restaurants and Home. Trans. Chin. Soc. Agric. Eng..

[6] Lu J., Peng K., Wang Q., Sun C. (2023). Lettuce Plant Trace-Element-Deficiency Symptom Identification via Machine Vision Methods. Agriculture.

[7] Ran J., Xi K., Xu K., Niu J. (2023). A quantum color image vector median filtering scheme. J. Quantum Electron..

[8] Wang Y., Li Q., Qi J. (2022). An image noise reduction method based on improved adaptive median filtering. Nav. Electron. Eng..

[9] Han Y., Wang X., Lu J. (2022). FPGA implementation of adaptive median filtering algorithm for real-time color images. Comput. Meas. Control.

[10] Stark J.A. (2000). Adaptive image contrast enhancement using generalizations of histogram equalization. IEEE Trans. Image Process..

[11] Lin S.C.F., Wong C.Y., Rahman M.A., Jiang G., Liu S., Kwok N., Shi H., Yu Y.-H., Wu T. (2015). Image enhancement using the averaging histogram equalization (AVHEQ) approach for contrast improvement and brightness preservation. Comput. Electr. Eng..

[12] Kim Y.-T. (1997). Contrast enhancement using brightness preserving bi-histogram equalization. IEEE Trans. Consum. Electron..

[13] Lee J., Pant S.R., Lee H.-S. (2015). An adaptive histogram equalization based local technique for contrast preserving image enhancement. Int. J. Fuzzy Log. Intell. Syst..

[14] Fang D., Fu Q., Wu A. (2023). Foggy sky image enhancement based on adaptive dynamic range CLAHE. Laser Optoelectron. Prog..

[15] Ju Z., Zhai C., Zhang W.X. (2021). A colorful merchandise label image segmentation method based on Chien SVM with region growing. Electron. Sci. Technol..

[16] Wang M., Lu A., Zhao W., Peng X., Long J., Huang S. (2019). An adaptive weld seam recognition algorithm based on improved Canny operator. Overseas Electron. Meas. Technol..

[17] Liu Y., Liu L., Liu Y. (2021). CT image-based intracranial hematoma detection and segmentation. Chin. J. Med. Phys..

[18] Fu W.B., He X., Yu J.Y. (2020). Combination of C-Canny algorithm and improved single-layer neural network for facial feature point localization. Comput. Eng. Sci..

[19] Upadhyay A., Chandel N.S., Singh K.P., Chakraborty S.K., Nandede B.M., Kumar M., Subeesh A., Upendar K., Salem A., Elbeltagi A. (2025). Deep learning and computer vision in plant disease detection: A comprehensive review of techniques, models, and trends in precision agriculture. Artif. Intell. Rev..

[20] Charisis C., Argyropoulos D. (2024). Deep learning-based instance segmentation architectures in agriculture: A review of the scopes and challenges. Smart Agric. Technol..

[21] Lei L., Yang Q., Yang L., Shen T., Wang R., Fu C. (2024). Deep learning implementation of image segmentation in agricultural applications: A comprehensive review. Artif. Intell. Rev..

[22] Shoaib M., Sadeghi-Niaraki A., Ali F., Hussain I., Khalid S. (2025). Leveraging deep learning for plant disease and pest detection: A comprehensive review and future directions. Front. Plant Sci..

[23] Liu K., Bouazizi M., Xing Z., Ohtsuki T. (2025). A Comparison Study of Person Identification Using IR Array Sensors and LiDAR. Sensors.

[24] Sumaiya I., Nasim R., Milon C. (2024). Detection and segmentation of lettuce seedlings from seedling-growing tray imagery using an improved mask R-CNN method. Smart Agric. Technol..

[25] Chowdhury M., Reza M.N., Jin H., Islam S., Lee G.-J., Chung S.-O. (2024). Defective Pennywort Leaf Detection Using Machine Vision and Mask R-CNN Model. Agronomy.

[26] Hoagland D.R., Arnon D.I. (1938). The water-culture method for growing plants without soil. Calif. Agric. Exp. Stn. Circ..

